# Risk Factors Affecting the Severity, Mortality, and Intensive Care Unit Admission of COVID-19 Patients: A Series of 1075 Cases

**DOI:** 10.3390/v17030429

**Published:** 2025-03-17

**Authors:** Ecem Narin Çopur, Dilek Ergün, Recai Ergün, Serap Atik, Hatice Türk Dağı, Muslu Kazım Körez

**Affiliations:** 1 Department of Pulmonary Medicine, Dr. Yaşar Eryılmaz Doğubeyazıt State Hospital, Ağrı 04402, Turkey; 2 Department of Pulmonary Medicine, Faculty of Medicine, Selcuk University, Konya 42130, Turkey; dilek.ergun@selcuk.edu.tr; 3 Department of Pulmonary Medicine, Iğdır Dr. Nevruz Erez State Hospital, Iğdır 76000, Turkey; atikserap.94@gmail.com; 4 Department of Medical Microbiology, Faculty of Medicine, Selcuk University, Konya 42130, Turkey; hturkdagi@selcuk.edu.tr; 5 Department of Biostatistics, Faculty of Medicine, Selcuk University, Konya 42130, Turkey; kkorez@selcuk.edu.tr

**Keywords:** COVID-19, disease severity, intensive care, mortality

## Abstract

Background: The clinical spectrum of severe acute respiratory syndrome coronavirus 2 (SARS-CoV-2) infection is broad; it can range from asymptomatic cases to mild upper respiratory tract illness, respiratory failure, and severe multiorgan failure resulting in death. Therefore, it is important to identify the clinical course of the disease and the factors associated with mortality. Objective: The aim of this study is to identify the risk factors associated with the severity of the disease, intensive care unit admission, and mortality in COVID-19 patients. Methods: A total of 1075 patients with clinical and radiological findings compatible with COVID-19 pneumonia and positive SARS-CoV-2 PCR were selected and retrospectively screened. All included patients were classified according to the 7th edition of the 2019 Coronavirus Disease Guidelines published by the National Health Commission of China. Results: It was observed that elevated white blood count (WBC) increased the severity of COVID-19 by 3.26 times and the risk of intensive care unit (ICU) admission by 3.47 times. Patients with high D-dimer levels had a 91% increased risk, and those with high fibrinogen levels had a 2.08 times higher risk of severe disease. High C-reactive protein (CRP) values were found to increase disease severity by 6.89 times, mortality by 12.84 times, and ICU admission by 3.37 times. Conclusions: Identifying the factors associated with disease severity, ICU admission, and mortality in COVID-19 patients could help reduce disability and mortality rates in pandemics.

## 1. Introduction

The clinical spectrum of severe acute respiratory syndrome coronavirus 2 (SARS-CoV-2) infection is broad; it can range from asymptomatic cases to mild upper respiratory tract illness, respiratory failure, and severe multiorgan failure resulting in death [1]. To guide early diagnosis and treatment of COVID-19 and predict morbidity and mortality, a detailed evaluation of demographic, clinical, laboratory, and imaging findings is required. There are currently no large retrospective case series in our country that provide data on the clinical, laboratory, radiological, and prognostic aspects of COVID-19. The aim of our study, which includes a large case series (*n* = 1075), is to identify the factors associated with mortality and prognosis based on the clinical, laboratory, and radiological findings of COVID-19 patients.

## 2. Materials and Methods

Our study is a single-center, retrospective, cross-sectional, descriptive observational study. A total of 1075 patients who were followed up in the Chest Diseases Intensive Care Unit and inpatient clinic with clinical and radiological findings compatible with COVID-19 pneumonia and a positive SARS-CoV-2 polymerase chain reaction (PCR) test between 2020 and 2021 were included. All included patients were classified according to the 2019 Coronavirus Disease Guidelines (7th edition) published by the National Health Commission of China. The classification is as follows: mild type: mild clinical symptoms without pneumonia on imaging; common type: pneumonia on imaging, along with fever, respiratory, and other symptoms; severe type: respiratory distress, respiratory rate ≥ 30/min at rest, oxygen saturation ≤ 93%, PaO_2_/FiO_2_ ≤ 300 mmHg; critical type: respiratory failure requiring mechanical ventilation, shock, and other organ failure requiring ICU monitoring and treatment [2].The first and second groups included patients classified as the non-severe group, while the third and fourth groups included patients classified as the severe group.

### 2.1. Statistical Analyses

All statistical analyses were performed using R version 3.6.0 (The R Foundation for Statistical Computing, Vienna, Austria; https://www.r-project.org, accessed on 1 January 2020). Before the analyses, the normality of the data was assessed using the Shapiro–Wilk normality test and Q-Q plots, while the homogeneity of group variances was checked using Levene’s test. Findings for numerical data are presented as mean ± standard deviation, and findings for categorical variables are presented as frequency (n) and percentage (%). The effects of demographic characteristics, comorbidities, medication use, disease symptoms, and radiological and clinical findings on disease severity, in-hospital mortality, and ICU admission were investigated using the Aspin–Welch *t*-test, Pearson’s chi-square test, Yates’ continuity-corrected chi-square test, and Fisher’s exact test. Furthermore, the effects of laboratory findings on disease severity, in-hospital mortality, and ICU admission were examined using multiple logistic regression models. When assessing the impact of laboratory findings on disease severity, adjusted odds ratios (adjusted OR) with 95% confidence intervals were calculated by adjusting for potential confounders, such as age, male gender, and comorbidities. Similarly, when examining the effects of laboratory findings on ICU admission, adjusted odds ratios (adjusted OR) with 95% confidence intervals were calculated by adjusting for potential confounders such as age, male gender, and comorbidities. On the other hand, when assessing the effects of laboratory findings on in-hospital mortality, adjusted odds ratios (adjusted OR) with 95% confidence intervals were calculated by adjusting for potential confounders such as age and comorbidities. A significance level of 5% was considered for the evaluation of all statistical hypotheses.

### 2.2. Ethical Approval

The study was approved by the Local Ethics Committee of Selçuk University Faculty of Medicine (no. 2021/194; dated 7 April 2021). Due to the retrospective design of the study, informed consent was waived.

## 3. Results

A total of 1075 patients, aged 17 to 99 years (57.78 ± 15.68), including 575 men (53.5%) and 500 women (46.5%), who were treated at Selçuk University Faculty of Medicine Hospital between March 2019 and August 2021, were included in the study. The average age of male patients was 57.81 ± 15.72 (range 17–93), and the average age of female patients was 57.75 ± 15.65 (range 20–99). Forty-two percent of the patients (*n* = 451) had no underlying disease, while 58% (*n* = 624) had a diagnosed medical condition. Of the 1075 patients followed, 17% (*n* = 187) were admitted to the ICU, and the in-hospital mortality rate was 10% (*n* = 108). The demographic, clinical, and radiological data of the patients are summarized in Table 1.

A 1-year increase in age was found to be associated with a 4% higher risk of disease severity, a 5% higher risk of mortality, and a 3% higher risk of ICU admission. Men had a 35% higher risk of severe disease and a 57% higher risk of ICU admission compared to women. However, there was no significant difference in mortality between the genders (*p* = 0.095). The presence of comorbidities increased the risk of severe disease by 2.68 times, the risk of mortality by 2.22 times, and the risk of ICU admission by 2.19 times.

Statistical analysis of the laboratory parameters in our study identified specific values associated with disease severity, mortality, and ICU admission. For disease severity and ICU admission, adjustments were made for gender, age, and comorbidities, while adjustments for mortality were based on age and comorbidities. The results are presented in Table 2, Table 3 and Table 4.

An increase in white blood count (WBC) was observed to raise the severity of COVID-19 by 3.26 times and the risk of ICU admission by 3.47 times (*p* < 0.001). In patients with a low platelet (PLT) count, the mortality risk was 2.03 times higher. Patients with a high RDW had a 64% higher risk of experiencing severe disease and a 42% higher risk of mortality. High neutrophil count and low lymphocyte count were associated with a significant increase in the severity, mortality, and risk of ICU admission in COVID-19 patients (*p* < 0.05). A high monocyte count increased the risk of severe disease by 61% and mortality by 88%, while it raised the ICU admission risk by 2.58 times. In patients with low eosinophil counts, the risk of experiencing severe disease was 2.05 times higher.

Low albumin and total protein levels showed a significant difference in the group with severe disease and the group admitted to the ICU (*p* < 0.001). Elevated lactate dehydrogenase (LDH) increased the risk of experiencing severe disease by 3.11 times, the risk of mortality by 2.62 times, and the risk of ICU admission by 2.56 times. Among liver function tests, elevated aspartate aminotransferase (AST) showed a significant difference in the severe disease group, the in-hospital death group, and the ICU admission group (*p* < 0.001), while there was no significant difference for alanine aminotransferase (ALT) levels between the groups. Low sodium (Na) levels were found to have a significant effect on disease severity (*p* < 0.001). Patients with low calcium (Ca) levels showed increased disease severity and a higher risk of ICU admission (*p* < 0.05). Creatinine levels were not associated with disease severity, ICU admission risk, or mortality (*p* > 0.05); however, high urea levels were found to increase mortality by 67% and the risk of ICU admission by 82%.

Elevated troponin levels were found to be associated with disease severity, ICU admission, and mortality (*p* < 0.05). High ferritin levels were found to be associated with the risk of severe disease by 80%, ICU admission by 97%, and mortality by 2.07 times. Patients with high D-dimer levels had a 91% increased risk (*p* < 0.05), and patients with high fibrinogen levels had 2.08 times the risk of experiencing severe disease.

High levels of C-reactive protein (CRP), one of the commonly used acute-phase reactants, were associated with a 6.89-fold increase in disease severity, a 12.84-fold increase in mortality, and a 3.37-fold increase in ICU admission. Patients with high procalcitonin levels had a 3.17-fold higher mortality rate and were 2.60 times more likely to be admitted to the ICU.

## 4. Discussion

A total of 1075 patients, including 575 men (53.5%) and 500 women (46.5%), aged between 17 and 99 years (57.78 ± 15.68), were included in our study. The average age of male patients was 57.81 ± 15.72 (17–93), while the average age of female patients was 57.75 ± 15.65 (20–99). Forty-two percent of the patients (*n* = 451) had no underlying disease, while 58% (*n* = 624) had a diagnosed disease. Among the 1075 patients followed, 17% (*n* = 187) were admitted to the ICU, and the hospital mortality rate was 10% (*n* = 108).

In a meta-analysis, the average age was 46.7, and 51.8% were male [3]. In an epidemiological study conducted in China, 51.0% of the patients were male [4]. In our study, 53% were male (*n* = 575), which is similar to the literature, and the average age was 57.78 ± 15.68, representing an older population. Forty-two percent of the patients (*n* = 451) had no underlying disease, while 58% (*n* = 624) had a diagnosed disease. In a study analyzing 352 critically ill COVID-19 patients, the mortality rate was found to be 32.1% (*n* = 113) [5], while in our study, the mortality rate was lower, at 10% (*n* = 108). It has been reported that the risk of COVID-19 mortality is higher in older individuals (>60 years) and in men [6]. Older patients are more prone to chronic diseases associated with severe COVID-19, but the excessive induction of pro-inflammatory cytokines may also be age-related [7], which increases the risk of acute lung injury [8].

In a meta-analysis including 1457 severe COVID-19 patients, 60% of the severe COVID-19 patients were male, 25% were over the age of 65, and 55% had comorbidities [9]. In one study, older age and a high number of comorbidities were associated with higher disease severity and mortality, and male gender was associated with more severe disease progression [10]. In our study, for each year increase in age, the severity of the disease was 4% higher, mortality was 5% higher, and ICU admission was 3% higher. The risk of severe disease was 35% higher in men compared to women, and the risk of ICU admission was 57% higher. In a cohort study conducted in Italy, multivariable analysis showed that a 10-year increase in age and male gender were significantly associated with mortality [11]. However, in our study, there was no significant relationship between gender and mortality, while male gender was closely related to ICU admission and disease severity.

In our study, the in-hospital mortality rate among COVID-19 patients was found to be 10% (*n* = 108). This rate is significantly lower compared to the rates reported in many other studies. For instance, a meta-analysis conducted by Zhou et al. reported an overall mortality rate of approximately 17% among hospitalized COVID-19 patients, with the rate rising to 32.1% in critically ill patients [12]. Similarly, Richardson et al., in a large cohort study conducted in New York, found a mortality rate of 21% among 5700 hospitalized COVID-19 patients [13]. Another study by Grasselli et al. in Italy reported a mortality rate of 26% among critically ill patients admitted to the intensive care unit (ICU) [14]. In our country, COVID-19 treatment, prevention, and vaccination programs were carried out according to the Ministry of Health guidelines [15]. These guidelines contributed to low mortality rates (10%) thanks to standard treatment protocols (e.g., corticosteroids, anticoagulants), early diagnosis, isolation strategies, and vaccination efforts. This rate is lower than the 17–32% range reported in other studies [12,13,14]. This difference can be explained by the relatively young population (mean age 57.78 ± 15.68), advanced treatment protocols, sufficient health system capacity, and strict public health measures [15]. In addition, it is thought that genetic and immunological factors may have affected clinical outcomes, and the reasons for regional mortality differences should be investigated [16].

Previous studies have shown that mortality rates varied significantly across different waves of the pandemic. Mortality was higher during the initial wave but decreased in subsequent waves due to factors such as improved treatment protocols, adaptation of healthcare systems, and increased clinical experience. Age and comorbidities remained consistent risk factors for mortality. These findings highlight the importance of dynamically evolving clinical management and treatment strategies during the pandemic [17].

In our study, we evaluated patients treated at our hospital between March 2020 and August 2021 as a whole. Had we analyzed patients separately according to the different waves of the pandemic, we could have more clearly demonstrated changes in mortality rates and the impact of evolving clinical management and treatment strategies on mortality over time. This represents a limitation of our study.

In a study by Wang and colleagues, which included 98 patients, it was shown that an increase in RDW could be a prognostic factor in critically ill patients. No significant differences were found between non-severe and critically ill patients for other routine hematological parameters, including WBC, neutrophils, monocytes, eosinophils, and PLT in the statistical analysis [18]. In a study analyzing 622 cases, RDW was elevated in 53% of those who did not survive and 43% of those who survived [19]. In our study, patients with a high RDW had more severe disease and, as a result, higher mortality.

In a retrospective study by Qin and colleagues, which diagnosed severe disease in 286 out of 452 COVID-19 patients, it was found that severe cases tended to have lower lymphocyte counts, higher leukocyte counts, and also lower percentages of monocytes, eosinophils, and basophils [20]. In a meta-analysis evaluating risk factors for mortality, it was found that in patients with severe and fatal disease, the WBC was significantly higher, and lymphocyte and platelet counts were lower compared to patients with non-severe disease and survivors [21]. In our study, elevated WBC was observed to increase the severity of COVID-19 by 3.26 times and the risk of ICU admission by 3.47 times. However, no relationship was found between elevated WBC and mortality. Other studies [22,23] have emphasized the relationship between lymphopenia and the need for ICU care. A meta-analysis found that elevated lymphopenia and neutrophil counts at the time of admission were significantly associated with an increased likelihood of severe disease progression and death [24]. In our study, high neutrophil count and low lymphocyte count were associated with a significant increase in the severity of COVID-19, mortality, and the risk of ICU admission (*p* < 0.05), which is consistent with the literature. However, in contrast to the literature, it was found that a high monocyte count, rather than a low monocyte count, increased the risk of severe disease by 61% and the risk of ICU admission by 2.58 times. Additionally, a high monocyte count was also found to be associated with mortality. In patients with low eosinophil counts, the risk of experiencing severe disease was 2.05 times higher.

In a meta-analysis, thrombocytopenia was found to be significantly associated with the severity of COVID-19, although there were differences between studies; in particular, a greater decrease in platelet count was observed in those who did not survive [25]. According to the findings of our study, patients with low PLT counts had a 2.03 times higher risk of mortality compared to patients with normal PLT counts. However, a low PLT count was not significantly associated with disease severity or the risk of ICU admission. In a case series examined by Wang and colleagues, in line with our findings, no significant difference was reported between ICU and non-ICU patients regarding platelets and monocytes [26]. In a 2020 meta-analysis, elevated ALT was found in 39.58% of severe cases and AST elevation was found in 49.68% of severe cases. Abnormal albumin levels were detected in 75.91% of severe cases and 31.04% of non-severe cases across studies [27].

It has been suggested that liver transaminases increase with SARS-CoV-2 infection. Significantly reduced albumin levels are common in severe COVID-19 cases, but changes in albumin are not parallel to the severity of hepatocellular damage in COVID-19 [28]. Hypoalbuminemia is common in many inflammatory diseases because increased capillary permeability can lead to the leakage of albumin into the interstitial space [29]. Other proposed mechanisms include the direct effect of the virus on hepatocytes or biliary epithelium, liver damage related to cytokine storms and immune-mediated injury, drug toxicity, and ischemic effects. In a case series, hepatitis, which can occur in patients with hemodynamic instability and multiple organ dysfunction [30], was more commonly observed in severe COVID-19 cases compared to non-severe cases, with liver function abnormalities such as hypoalbuminemia, aminotransferases, and bilirubin levels. Elevations in ALT and AST were similar in general COVID cases, but the prevalence of AST elevations was higher than ALT in severe COVID cases [27]. In a case series, patients with hypoalbuminemia tended to be older, had a longer onset period, and often required intensive care treatment. Additionally, in patients with hypoalbuminemia, a higher mortality rate was observed (13.2%) compared to the normal albumin group (1%), and this was concluded to be an independent predictive factor for mortality [31]. In a retrospective study of 609 patients, hypoalbuminemia was observed as an early indicator of in-hospital mortality in COVID-19 patients, independent of age, comorbidities, and inflammatory markers [32].

According to the results of our study, similar to the literature, low albumin and total protein levels showed a significant difference between the group with severe disease and the group admitted to the intensive care unit. Elevated LDH increased the risk of severe disease by 3.11 times, the risk of fatal progression by 2.62 times, and the risk of ICU admission by 2.56 times. However, in contrast to the literature, low albumin was not closely associated with mortality, while low total protein was closely related to it. Among the liver function tests, elevated AST showed a significant difference in the severe disease group, the in-hospital death group, and the ICU admission group, whereas in contrast to the literature, no significant difference was found in any group for elevated ALT.

Hypocalcemia is quite prominent in COVID-19 and is reported in 60% or more of hospitalized patients [33]. In a study evaluating risk factors, hypocalcemia was found to be associated with prolonged hospital stays [34], the need for ventilatory support, ICU admission, and mortality [35]. Therefore, the degree of hypocalcemia represents a strong measure of disease severity, and ca supplementation has been recommended [36]. In our study, patients with low calcium (Ca) levels were found to have increased disease severity and a higher risk of ICU admission (*p* < 0.05). This finding is consistent with recent studies suggesting that hypocalcemia is a common electrolyte disorder in COVID-19 patients and may serve as a marker of disease severity. Sun et al. demonstrated that hypocalcemia is prevalent in COVID-19 patients and is associated with worse clinical outcomes, including increased disease severity and mortality [37]. Similarly, Cappellini et al. reported that low total and ionized calcium levels are frequently observed in COVID-19 patients, further supporting the role of calcium as a potential prognostic marker [38].

Hypocalcemia in COVID-19 can be attributed to multiple factors. Li et al., in their systematic review and meta-analysis, emphasized that hypocalcemia is significantly associated with severe COVID-19, likely due to the systemic inflammatory response and cytokine storm, which may disrupt calcium homeostasis [39]. Additionally, vitamin D deficiency, which is common in critically ill patients, may contribute to hypocalcemia, as vitamin D plays a crucial role in calcium absorption and regulation [40]. In our cohort, the presence of chronic kidney disease (CKD) in 62 patients likely exacerbated this issue, as CKD is often associated with secondary hyperparathyroidism and reduced renal activation of vitamin D, both of which can lead to hypocalcemia [33].

Furthermore, medications commonly used in the treatment of COVID-19, such as corticosteroids and antivirals, may also affect calcium levels. These findings collectively highlight the multifactorial nature of hypocalcemia in COVID-19 and its potential role as a prognostic marker. Monitoring and correcting calcium levels in COVID-19 patients, especially those with underlying comorbidities such as CKD, may be crucial for improving clinical outcomes.

In one study, higher white blood cell and neutrophil counts, as well as elevated levels of D-dimer, creatine kinase, and creatinine, were observed in patients admitted to the ICU [23]. Studies have concluded that hyponatremia may be an indicator of poor prognosis. For example, it has been shown that in COVID-19 patients with hyponatremia, disease severity, hospital admission, ICU transfer, use of ventilation, and mortality rates were higher compared to COVID-19 patients with normonatremia [41,42]. In severe cases, it has been suggested that early kidney damage in COVID-19 patients could often lead to hypokalemia and hyponatremia. This could also be linked to an increased release of antidiuretic hormone in response to volume depletion following gastrointestinal fluid loss [43]. In our study, hyponatremia in severe COVID-19 patients was found to be an indicator of disease severity and ICU admission, which is consistent with the literature [42,44]. However, we found that creatinine levels were not associated with disease severity, ICU admission, or mortality (*p* > 0.05). In contrast, elevated urea levels were significantly associated with a 67% increase in mortality and an 82% increase in the risk of ICU admission. The lack of a significant association between creatinine levels and outcomes could be explained by factors such as population differences or preserved renal function in most patients.

A study has shown that D-dimer, fibrinogen, prothrombin time (PT), and activated partial thromboplastin time (aPTT) can be effectively used in assessing the severity and prognosis of the disease [45]. In a study evaluating 28-day mortality, D-dimer and PT were reported to have a positive correlation with mortality [46]. In our study, elevated levels of troponin and ferritin predicted the severity, mortality, and ICU admission of COVID-19 patients. Since age and D-dimer are positively correlated, it is unclear whether adjustments were made in other studies. In our study, after adjusting for age and comorbidities, no association with mortality was found for D-dimer.

The severity of COVID-19, according to the literature, is most significantly associated with high levels of CRP, LDH, WBC, and RDW and the presence of lymphopenia and eosinopenia. According to the literature, risk factors for ICU admission in COVID-19 patients include high WBC, elevated neutrophil count, and lymphopenia, while no significant relationship was found with low PLT and high D-dimer. The parameters most affecting mortality in COVID-19 patients are CRP, procalcitonin, troponin, ferritin, aPTT, RDW, and high monocyte levels.

This study has some limitations. It has a single-center and retrospective design, which limits the generalizability of the findings. Due to the retrospective design, there may be missing or inaccurate data, and establishing causal relationships is challenging. Additionally, treatment protocols and follow-up processes may not have been standardized, which could have affected the interpretation of the results. Considering these limitations, the findings should be interpreted with caution and supported by more comprehensive studies in the future.

Future studies should be designed as multicenter and prospective to overcome these limitations. Multicenter studies will enhance the generalizability of findings by collecting data from diverse geographic regions and patient populations. Additionally, prospective studies allow for a more controlled and standardized data collection process, enabling clearer establishment of causal relationships. Especially in a dynamic and rapidly evolving pandemic like COVID-19, multicenter and prospective studies are crucial for evaluating the effectiveness of treatment protocols and developing new therapeutic strategies. Such studies will not only guide clinical practice but also make significant contributions to the literature.

## 5. Conclusions

In conclusion, our study, which represents the largest case series in our country, systematically identifies risk factors for COVID-19 and provides detailed demographic, clinical, radiological, and laboratory findings. While this comprehensive analysis offers valuable insights into the course of the disease, mortality, ICU admission, and severity within our national context, we acknowledge that the single-institution design may limit the generalizability of our results to other geographic regions. Nevertheless, we believe that our findings make a significant contribution to the literature by providing a robust foundation for understanding COVID-19 in our country. Future multicenter studies involving diverse populations are needed to validate and extend these findings, enabling broader applicability and more comprehensive insights into the disease.

## Figures and Tables

**Table 1:** Demographic characteristics of the patients.

	Non-Severe (*n* = 553)	Severe (*n* = 522)	Total (*n* = 1075)	*p*-Value
Demographic Characteristics				
Age	53.26 ± 15.84	62.57 ± 14.01	57.78 ± 15.68	<0.001 ^1^
Gender (Male/Female)	276/277	299/223	575/500	0.015 ^2^
Comorbidities (n%)	258 (41.3)	366 (58.7)	624 (58)	<0.001 ^2^
Diabetes Mellitus	95 (40.3)	141 (59.7)	236 (22)	<0.001 ^2^
Hypertension	121 (39.8)	183 (60.2)	304 (28.3)	<0.001 ^2^
Coronary Artery Disease	42 (30.9)	94 (69.1)	136 (12.7)	<0.001 ^2^
Respiratory Disease	33 (29.7)	78 (70.3)	111 (10.3)	<0.001 ^2^
Malignancy	31 (34.1)	60 (65.9)	91 (8.5)	<0.001 ^2^
Chronic Kidney Disease	21 (33.9)	41 (66.1)	62 (5.8)	0.004 ^2^
Presence of Symptoms (n%)	493 (49)	514 (51)	1007 (93.7)	<0.001 ^2^
Fever	120 (47.4)	133 (52.6)	253 (23.5)	0.144 ^2^
Cough	273 (51.4)	258 (48.6)	531 (49.4)	0.985 ^2^
Shortness of Breath	173 (32.3)	362 (67.7)	535 (49.8)	<0.001 ^2^
Sputum	27 (49.1)	28 (50.9)	55 (5.1)	0.720 ^2^
Sore Throat	48 (58.5)	34 (41.5)	82 (7.6)	0.181 ^2^
Headache	58 (65.2)	31 (34.8)	89 (8.3)	0.007 ^2^
Weakness	143 (53.6)	124 (46.4)	267 (24.8)	0.425 ^2^
Diarrhea	32 (68.1)	15 (31.9)	47 (4.4)	0.029 ^3^
Muscle and Joint Pain	142 (60.4)	93 (39.6)	235 (21.9)	0.002 ^2^
Abdominal Pain	8 (57.1)	6 (42.9)	14 (1.3)	0.873 ^3^
Nausea/Vomiting	19 (65.5)	10 (34.5)	29 (2.7)	0.177 ^3^
Chest Pain	4 (30.8)	9 (69.2)	13 (1.2)	0.222 ^3^
Hemoptysis	3 (37.5)	5 (62.5)	8 (0.7)	0.495 ^4^
Loss of Taste/Smell	23 (79.3)	6 (20.7)	29 (2.7)	0.004 ^3^
Radiological Findings (n%)				
Bilateral Ground Glass Opacity	428 (47.5)	474 (52.5)	902 (83.9)	<0.001 ^2^
Unilateral Ground Glass Opacity	71 (71.7)	28 (28.3)	99 (9.2)	<0.001 ^2^
Consolidation	100 (39.7)	152 (60.3)	252 (23.4)	<0.001 ^2^
Pleural Effusion	7 (20)	28 (80)	35 (3.3)	<0.001 ^2^
Atelectasis	51 (53.1)	45 (46.9)	96 (8.9)	0.730 ^2^
Fibrotic Bands	96 (42.7)	129 (57.3)	225 (20.9)	0.003 ^2^
Central Localization	75 (33.8)	147 (66.2)	222 (20.7)	<0.001 ^2^
Peripheral Localization	487 (49.6)	495 (50.4)	982 (91.3)	<0.001 ^2^
Pneumothorax/Pneumomediastinum	12 (52.2)	11 (47.8)	23 (2.1)	0.943 ^2^

The data are presented as mean ± standard deviation or frequency (n) and percentage (%). ^1^ Aspin–Welch *t*-test. ^2^ Pearson chi-square test. ^3^ Yates continuity correction chi-square test. ^4^ Fisher’s exact test. The *p*-value refers to the severe and non-severe groups.

**Table 2:** Factors associated with COVID-19 disease severity.

Parameters	Odds Ratio (OR)	Lower Limit	Upper Limit	*p*-Value
Demographic characteristics				
Age (years)	1.04	1.03	1.05	<0.001
Male gender (reference: female)	1.35	1.06	1.71	0.016
Presence of comorbidities (reference: none)	2.68	2.09	3.45	<0.001
Laboratory findings *				
WBC (reference: 3.5–10.5 K/uL)				
>10.5 K/uL	3.26	2.13	5.01	<0.001
RDW (refrence: %11.8–%15.5) >%15.5	1.64	1.17	2.31	0.004
Neutrophil (reference: 1.7–7 K/uL)				
>7 K/uL	3.09	2.18	4.38	<0.001
Lymphocyte (reference: 0.90–2.90 K/uL)				
<0.90 K/uL	1.63	1.23	2.15	<0.001
Monocyte (reference: 0.20–0.80 K/uL)				
>0.80 K/uL	1.61	1.07	2.44	0.023
Eosinophil (reference: 0.05–0.50 K/uL) <0.05 K/uL	2.05	1.46	2.87	<0.001
Albumin (reference: 3.5–5.2 g/dL) <3.5 g/dl	2.94	2.22	3.88	<0.001
Total protein (reference: 6.6–8.7 g/dL) <6.6 g/dL	1.83	1.40	2.39	<0.001
AST (reference: 0–40 U/L) >40 U/L	2.20	1.70	2.86	<0.001
LDH (reference: 135–214 U/L)				
>214 U/L	3.11	2.18	4.43	<0.001
Na (reference: 136–145 mEq/L)				
<136 mEq/L	1.67	1.28	2.19	<0.001
Ca (reference: 8.6–10.5 mg/dL)				
<8.6 mg/dl	2.10	1.62	2.74	<0.001
Troponin (reference: 0–14 ng/L) >14 ng/L	1.64	1.13	2.38	0.009
Ferritin (reference: 30–400 ng/mL)				
>400 ng/ml	1.80	1.34	2.40	<0.001
D-dimer (reference: 0–500 ng/mL) >500 ng/ml	1.91	1.46	2.50	<0.001
Fibrinogen (reference: 200–500 mg/dL) >500 mg/dL	2.08	1.51	2.88	<0.001
CRP (reference: 0–5 mg/dL) >5 mg/dl	6.89	4.03	11.78	<0.001

* Adjustment has been made for age, gender, and comorbidities. Abbreviations: WBC, white blood count; RDW, red cell distribution width; AST, aspartate aminotransferase; LDH, lactate dehydrogenase; Na, sodium; Ca, calcium; CRP, C-reactive protein.

**Table 3:** Factors associated with COVID-19 mortality.

Parameters	Odds Ratio (OR)	Lower Limit	Upper Limit	*p*-Value
Demographic characteristics				
Age (years)	1.05	1.04	1.07	<0.001
Male gender (reference: female)	1.41	0.94	2.12	0.095
Presence of comorbidities (reference: none)	2.22	1.42	3.48	<0.001
Laboratory findings *				
Platelet (reference: 150–450 K/uL)				
<150 K/uL	2.03	1.31	3.15	0.002
RDW (refrence: %11.8–%15.5) >%15.5	2.76	1.76	4.31	<0.001
Neutrophil (reference: 1.7–7 K/uL)				
>7 K/uL	1.90	1.21	2.98	0.005
Lymphocyte (reference: 0.90–2.90 K/uL)				
<0.90 K/uL	2.53	1.67	3.85	<0.001
Monocyte (reference: 0.20–0.80 K/uL)				
>0.80 K/uL	1.88	1.09	3.26	0.024
Total protein (reference: 6.6–8.7 g/dL) <6.6 g/dl	1.77	1.12	2.79	0.015
AST (reference: 0–40 U/L) >40 U/L	1.64	1.08	2.48	0.020
LDH (reference: 135–274 U/L)				
>274 U/L	2.62	1.28	5.34	0.008
Urea (reference: 16.6–48.5 mg/dL)				
>48.5 mg/dl	1.67	1.07	2.61	0.025
Troponin reference: 0–14 ng/L) >14 ng/L	2.92	1.83	4.67	<0.001
Procalcitonin (reference: 0–0.5 ug/L) >0.5 ug/L	3.17	1.81	5.56	<0.001
Ferritin (reference: 30–400 ng/mL)				
>400 ng/ml	2.07	1.37	3.14	<0.001
aPTT (reference: 25–40)				
>40	3.39	1.33	8.61	0.010
CRP (reference: 0- 5 mg/dL) >5 mg/dl	12.84	1.76	93.40	0.012

* Adjustment has been made for age and comorbidities. Abbreviations: RDW, red cell distribution width; AST, aspartate aminotransferase; LDH, lactate dehydrogenase; aPTT, activated partial thromboplastin time; CRP, C-reactive protein.

**Table 4:** Factors associated with COVID-19 intensive care unit admission.

Parameters	Odds Ratio (OR)	Lower Limit	Upper Limit	*p*-Value
Demographic characteristics				
Age (years)	1.03	1.02	1.04	<0.001
Male gender (reference: female)	1.57	1.13	2.17	0.006
Presence of comorbidities (reference: none)	2.19	1.54	3.10	<0.001
Laboratory findings *				
WBC (reference: 3.5–10.5 K/uL)				
>10.5 K/uL	3.47	2.32	5.19	<0.001
RDW (refrence: %11.8–%15.5) >%15.5	1.74	1.18	2.55	0.005
Neutrophil (reference: 1.7–7 K/uL)				
>7 K/uL	3.26	2.28	4.67	<0.001
Lymphocyte (reference: 0.90–2.90 K/uL)				
<0.90 K/uL	2.24	1.61	3.14	<0.001
Monocyte (reference: 0.20–0.80 K/uL)				
>0.80 K/uL	2.58	1.67	4.00	<0.001
Albumin (reference: 3.5–5.2 g/dL) <3.5 g/dl	2.33	1.64	3.32	<0.001
Total protein (reference: 6.6–8.7 g/dL) <6.6 g/dl	1.94	1.36	2.77	<0.001
AST (reference: 0–40 U/L) >40 U/l	1.48	1.07	2.04	0.019
LDH (reference: 135–274 U/L)				
>274 U/L	2.56	1.50	4.36	<0.001
Urea (reference: 16.6–48.5 mg/dL)				
>48.5 mg/dl	1.82	1.26	2.62	0.001
Ca (reference: 8.6–10.5 mg/dL)				
<8.6 mg/dl	2.39	1.67	3.42	<0.001
Troponin (reference: 0–14 ng/L) >14 ng/L	1.95	1.31	2.91	<0.001
Procalcitonin (reference: 0–0.5 ug/L) >0.5 ug/L	2.60	1.58	4.28	<0.001
Ferritin (reference: 30–400 ng/mL)				
>400 ng/ml	1.97	1.40	2.78	<0.001
CRP (reference: 0–5 mg/dL) >5 mg/dl	3.37	1.53	7.44	0.003

* Adjustment has been made for age, gender, and comorbidities. Abbreviations: WBC, white blood count; RDW, red cell distribution width; AST, aspartate aminotransferase; LDH, lactate dehydrogenase; Ca, calcium; CRP, C-reactive protein.

## Data Availability

The data presented in this study are available upon request from the corresponding author.

## Abbreviations

CKD	Chronic kidney disease
ALT	Alanine aminotransferase
aPTT	Activated partial thromboplastin time
AST	Aspartate aminotransferase
Ca	Calcium
CRP	C-reactive protein
ICU	Intensive care unit
LDH	Lactate dehydrogenase
Na	Sodium
PLT	Platelet
PT	Prothrombin time
RDW	Red cell distribution width
SARS-CoV-2	Severe acute respiratory syndrome coronavirus 2
WBC	White blood count
